# Fuse your mitochondria, lose appetite: an anorexic, anti‐obesity sphingolipid

**DOI:** 10.15252/emmm.202114618

**Published:** 2021-07-19

**Authors:** Carolin Muley, Alexander Bartelt

**Affiliations:** ^1^ Institute for Cardiovascular Prevention (IPEK) Ludwig‐Maximilians‐University Munich Germany; ^2^ German Center for Cardiovascular Research Partner Site Munich Heart Alliance Ludwig‐Maximilians University Munich Munich Germany; ^3^ Institute for Diabetes and Cancer (IDC) Helmholtz Center Munich German Research Center for Environmental Health Neuherberg Germany; ^4^ Department of Molecular Metabolism & Sabri Ülker Center Harvard T.H. Chan School of Public Health Boston MA USA

**Keywords:** Metabolism, Chemical Biology

## Abstract

Aberrant production of ceramides, the precursors of complex sphingolipids, is a hallmark of obesity and strongly linked to metabolic dysfunction (Meikle and Summers 2017). Ceramides are formed by recycling or de novo synthesis from sphingosine and a fatty acid side chain moiety. The side chain length determines lipotoxicity of ceramides, as those composed of C16:0 or C18:0 side chains are toxic whereas those with C24:0 or C24:1 are not (Meikle and Summers 2017). Counteracting the deleterious effects of high‐fat diets (HFDs) rich in saturated fat either by inhibiting synthesis or by promoting degradation of ceramides mitigates insulin resistance and ectopic lipid accumulation (Meikle and Summers 2017). However, drugs that safely and selectively target ceramide metabolism have failed to translate into metabolic benefit in human trials so far.

## Graphical abstract

A. Bartelt and C. Muley discuss the study by Jayashankar, Selwan *et al* (in this issue of *EMBO Mol Med*) that introduces an orally bioavailable synthetic sphingolipid that enhances mitochondrial fusion, leading to reduced food intake, weight loss, and improved metabolic health in obese mice.

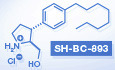

In this issue of *EMBO Molecular Medicine*, Jayashankar & Selwan *et al* show that the synthetic sphingolipid SH‐BC‐893 both prevents and cures diet‐induced obesity in mice by suppressing food intake (Jayashankar *et al,* 2021). The lipotoxicity of palmitate and its related ceramides is partly attributed to an imbalance in mitochondrial dynamics toward increased fission and fragmentation (Hammerschmidt *et al,* 2019). In search for an approach to counteract these lipotoxic effects, the authors tested several compounds reported to have beneficial effects in cultured cells, and all but one, SH‐BC‐893, failed to counteract palmitate and ceramide‐induced mitochondrial fragmentation. SH‐BC‐893 is a known synthetic sphingolipid, and the authors implicate SH‐BC‐893 in the trafficking of intracellular membranes, particularly in the recruitment of dynamin‐related protein‐1 (Drp1) to the outer mitochondrial membrane (Palmer *et al,* 2013). The exact mechanism of action of SH‐BC‐893 is still unclear. The authors report the involvement of SH‐BC‐893 in inhibition of both lipid kinase PIKfyve and ARF6 GTPase, which mediate endolysosomal trafficking and influence Drp1 recruitment. However, where SH‐BC‐893 actually “binds” remains unclear. More work will help understanding whether this is a specific inhibitory effect, interfering with the membrane trafficking machinery, or whether the impact of SH‐BC‐893 is related to membrane lipid metabolism and composition. Interestingly, while previous work has shown that ceramide‐induced mitochondrial fission also causes ER stress, treatment with SH‐BC‐893 blocks this effect, too. This indicates that—at least in fibroblasts—this mitochondrial remodeling is beneficial. Nevertheless, the balance of fission and fusion is dynamically regulated, and while some cell types display a rather fused network, in other cell types, e.g., in brown adipocytes, fission enhances mitochondrial respiration (Wikstrom *et al,* 2014). The effects of SH‐BC‐893 will need to be carefully dissected in more specialized cell types by an extended set of methods that will include assays of bioenergetics and mitochondrial quality control.

Based on the beneficial effects of SH‐BC‐893 in cultured cells, Jayashankar & Selwan *et al* further treated HFD‐induced obese mice with SH‐BC‐893. Oral SH‐BC‐893 application in mice with established obesity resulted in a remarkable drop in body weight, which was associated with improved glucose tolerance and reduced accumulation of fat in the liver despite continued feeding of HFD. A pair‐feeding study revealed that the beneficial effects of SH‐BC‐893 were mainly due to lowering food intake, which was already detectable after one dose of SH‐BC‐893. Surprisingly, energy expenditure remained unchanged, which is usually observed either when food intake is suppressed or during fasting. This raises several interesting questions of translational relevance. First, where in the body does the action of SH‐BC‐893 take place? The most obvious hypothesis is that SH‐BC‐893 treatment impacts on hypothalamic neurons that control energy metabolism, especially since ceramides have been implicated in regulating food intake in the hypothalamus (Contreras *et al,* 2014). In line with this, the authors demonstrate that neurons in the arcuate nucleus displayed a fused mitochondrial network after treatment with SH‐BC‐893. Mitochondrial dynamics mediated by mitofusin‐2 in POMC neurons are known to influence neuron sensitivity to leptin (Schneeberger *et al,* 2013). Indeed, one dose of SH‐BC‐893 was sufficient to lower plasma leptin levels, which suggests that another mechanism of leptin secretion is implicated, potentially stemming from SH‐BC‐893 action in adipocytes. Additionally, in the pair‐fed animals, leptin levels did not further improve, indicating that the drop in leptin drives rather than follows the weight reduction. Second, is SH‐BC‐893 truly a leptin sensitizer, or does it regulate leptin secretion from adipocytes, too? More work will be needed to determine whether leptin is required for SH‐BC‐893 to fulfill its therapeutic potential, for example, by using the *db*/*db* and *ob*/*ob* mouse models or by using leptin injections to evaluate whether leptin is able to counteract the effects of SH‐BC‐893 on food intake.

Another interesting aspect of the work by Jayashankar & Selwan relates to the peripheral effects of SH‐BC‐893. As mentioned above, one possible interpretation is that the effect on food intake also stems from leptin level lowering. Human mutations in *MFN2* lead to mitochondria being more fragmented and more cellular autophagosomes (Rocha *et al,* 2017), which indicates impaired mitophagy as balanced fission–fusion events are required for sustaining healthy mitochondria in adipocytes (Bartelt *et al,* 2018). However, in patient fibroblasts mitochondrial morphology and gene expression are normal, reinforcing the notion that the outcome of impaired mitochondrial dynamics is highly dependent on the cell type in question. In other words, SH‐BC‐893 treatment systemic outcome could be a combination of beneficial and deleterious effects of forcing a fused mitochondrial network. Albeit speculative, by using SH‐BC‐893, one might kill two birds with one stone—increase leptin sensitivity in the hypothalamus while decreasing leptin secretion from white adipocytes. Beyond leptin and adipocytes, ceramides and mitochondrial dynamics also have significant impact on liver and muscle function (Hammerschmidt *et al,* 2019), which might explain why SH‐BC‐893 did not concomitantly lead to reduced energy expenditure. Originally, SH‐BC‐893 was developed as an anti‐tumor agent and shown to specifically inhibit growth of *Ras*‐active tumor, by blocking both nutrient influx and lysosomal nutrient recycling via activation of protein phosphatase 2A (PP2A) (Kim *et al,* 2016). Jayashankar & Selwan *et al* confirm that SH‐BC‐893 inhibits mitochondrial fragmentation by inhibiting PIKfyve and ARF6, but whether the effect on energy metabolism involves PP2A activation remains unclear. Nevertheless, the creation of a fused mitochondrial network might simultaneously protect from certain obesity‐linked cancers.

Finally, what is the translational perspective of SH‐BC‐893 or other therapeutics targeting ceramides and mitochondria? The experiments were performed in mice on HFD, which is an accepted model for human obesity‐linked metabolic dysfunction. Considering that HFD consumption drives ceramide production, and particularly those with high cellular toxicity (Meikle & Summers, 2017), one might anticipate that diet will play an important role. Again, experiments in hyperphagic mouse models that become obese on regular diets might prove insightful. On a side note, Jayashankar & Selwan actually do show that SH‐BC‐893 treatment of regular mice on a chow diet triggers similar effects on mitochondria in hypothalamic neurons compared to those fed HFD. Somewhat paradoxically, while the food intake was lower in these mice, there was no body weight loss with the SH‐BC‐893 treatment, suggesting that SH‐BC‐893 may influence the balance of energy intake and energy expenditure only under certain circumstances that include obesity. In conclusion, the study by Jayashankar & Selwan illuminates the relevance of ceramide‐induced lipotoxicity and its impact on the coupling of energy intake and energy expenditure. SH‐BC‐893 might turn out as both an extremely useful tool for understanding molecular mitochondrial dynamics as well as paving the way for new sphingolipid‐derived anti‐obesity therapeutics (Fig 1).

**Figure 1:**
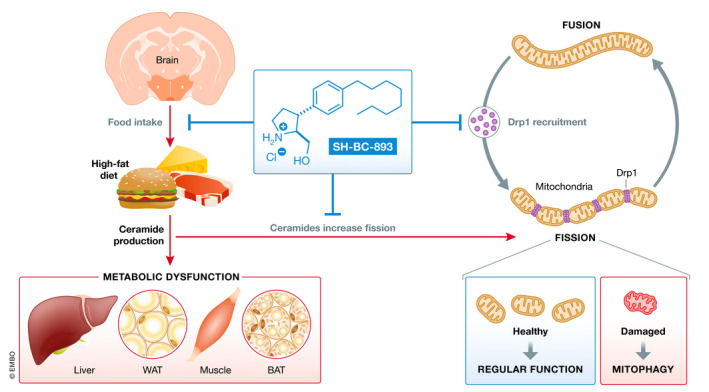
**SH‐BC‐893 is a synthetic sphingolipid that blocks ceramide‐induced mitochondrial fission and food intake**.
